# The impact of acute surgical unit rostering on National Emergency Access Targets during the COVID‐19 pandemic: a single hospital experience

**DOI:** 10.1111/ans.17498

**Published:** 2022-02-01

**Authors:** Matthew Corbitt, Jonathan H. Wiener, Kate Swift, Phuc (Richard) Do, Roxanne Wu

**Affiliations:** ^1^ Department of Surgery Cairns and Hinterland Hospital and Health Service Cairns Queensland Australia; ^2^ School of Medicine & Dentistry Griffith University Gold Coast Queensland Australia; ^3^ Faculty of Medicine University of Queensland St Lucia Queensland Australia; ^4^ College of Medicine and Dentistry James Cook University Cairns Queensland Australia

**Keywords:** acute surgical, coronavirus, COVID‐19, NEAT, outcomes, rostering

## Abstract

**Background:**

Surgical departments have been dramatically impacted by the novel coronavirus 19 (COVID‐19) pandemic, with the cancellation of elective cases and changes to the provision of emergency surgical care. The aim of this study was to determine whether structural changes made within our facility's surgical department during COVID‐19 altered National Emergency Access Target (NEAT) times and impacted on patient outcomes.

**Methods:**

Emergency surgical cases over a 4‐month time period were retrospectively collected and statistically analysed, divided into pre‐ and mid‐COVID‐19 pandemic.

**Results:**

Baseline characteristics between the groups were comparable. There was a significant increase in consultant presence in theatre in the COVID group. There were also statistically significant reductions in NEAT times at each timepoint, although these did not meet national guidelines. There was no change in emergency surgical workload, complication rate or mortality rates within 30 days.

**Conclusion:**

Any significant change to services requires a coordinated hospital‐wide approach, not just from a single department, and clinicians must continue to be wary of benchmarked times as the overall feasibility and safety of NEAT times has also been highlighted again.

## Graphical abstract

This study determined that after restructuring our surgical department to an ASU roster during mid‐COVID‐19, our National Emergency Access Target (NEAT) times improved, but not enough to meet national standards and there was no change in patient outcomes. Our results again indicate that any significant change to services requires a coordinated hospital‐wide approach, as well as highlight whether achieving NEAT times is actually feasible and safe.

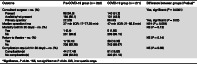

## Introduction

The outbreak of novel coronavirus disease 19 (COVID‐19) caused unprecedented challenges within global healthcare systems, with many of these challenges persisting into 2021. Effects have been widespread across training, administration and the provision of surgical care training, most notably by restricting services to emergency and category one cases only. Surgical units have also been restructured to include more telehealth,^1^ redirection away from emergency departments and greater use of non‐operative measures. Structural changes made during this time period offer a unique opportunity to reflect and improve our surgical department.

Cairns Hospital is the tertiary referral centre for Far North Queensland, with a population of 280 000 people inhabiting an area of 140 000 km^2^. In the height of the COVID‐19 pandemic, Cairns Hospital introduced an acute surgical unit (ASU) in accordance with general surgeons Australia (GSA) 12‐point plan for emergency general surgery.^2^ Additionally, a dedicated surgical rapid assessment unit (RAU) was created to improve emergency flow and redirect patients away from an overburdened emergency department.^4^ The goal was to reduce overlap and redundancy in rostering, increase the availability of senior surgical staff and reduce emergency department workload and possible COVID‐19 exposure for surgical patients and staff.

Our study aimed to investigate whether the emergency surgical workload at Cairns Hospital changed during COVID‐19, and whether the new ASU rostering and additional RAU resulted in improved flow as measured by adherence to the National Emergency Access Target (NEAT), and subsequently whether this impacted on patient outcomes.

## Methods

This was a single‐centre retrospective study conducted at Cairns Hospital, a large regional hospital servicing Far North Queensland, Australia. The consultant staff consisted of eight general surgeons and three vascular surgeons covering a 24 h, 7‐day per week on‐call roster for each surgical service. The vascular surgeons were not part of the general surgery on‐call roster. Data were collected and analysed for every patient admitted under a general surgical or vascular consultant that underwent an emergency surgical procedure during the same admission, between 1 February 2020 and 31 May 2020. Data were collected from a pre‐existing database managed by the theatre data coordinator and verified by chart review of the integrated electronic medical record (iEMR) and peri‐operative tracking records. This project was endorsed by the Far North Queensland HREC and deemed exempt from full Ethics Committee review (project reference LNR/2020/QCH/66686–1465 QA).

Patients were categorized into ‘pre‐COVID’ or ‘COVID’ groups based upon timing of their emergency surgical procedure. ‘Pre‐COVID’ included all patients operated on between 1 February 2020 to 31 March 2020, the pre‐existing surgical roster. During this roster for General Surgery, there was a dedicated general surgical consultant, registrar, and unaccredited registrar on‐site from 0700 to 2100, with one unaccredited surgical registrar on‐site overnight with a general surgical consultant on‐call. For vascular surgery, there was a vascular surgeon, registrar and unaccredited registrar on‐site from 0700 to 1600 with the unaccredited general surgical registrar covering after hours with a vascular surgeon on‐call.

‘COVID’ was defined as the new ASU surgical roster implemented on 1 April 2020. During this period, there was 24‐h on‐call cover provided by teams consisting of one on‐site surgical consultant and two on‐site surgical registrars (accredited or unaccredited), with each team covering 12‐h shifts. Additionally on weekdays, there was also a second‐team consisting of a surgical consultant, registrar and unaccredited registrar available on‐site from 0700 to 1600. Implementation of weekday access to a surgical RAU located in the vacant surgical outpatient area, separate from the main Emergency Department, was also created. There was no sub‐specialty roster for either group, with both rosters having access to a 24‐h emergency operating theatre, although, restricted operating after hours existed for the pre‐COVID roster.

All consultant surgeons are full‐time staff specialists, and it was hospital policy during COVID rostering that where possible, the surgical consultant must be the primary operator to help reduce theatre case time. Shift times were also increased, with overall fortnightly hours remaining the same, to decrease contact between teams in case of a COVID outbreak.

Patient demographics (age, sex, anaesthetic classification), hospital length of stay (LOS), emergency department NEAT times (triage time, timing of investigations, time to referral, time to admission), operation details (procedure undertaken, booking priority category, seniority of primary operator, length of surgery as determined by surgical start/stop times, in‐hours versus after‐hours start time, and surgical complications and mortality at 30‐days) were recorded.

Patients who underwent an elective surgery, were admitted under a different specialty (other than general surgery or vascular surgery), or did not undergo a surgical procedure during their admission were excluded. Any patients who underwent multiple emergency operations during the same admission had data collected from their first/index operation, and all subsequent surgeries were tallied. Any patient who presented multiple times over the study period had each admission included as a separate entry.

Data are reported as a number (percentage) for categorical data and mean (*SD*) or median (inter‐quartile range, IQR) for continuous data according to normal or non‐normal distribution. Normality was determined by visual inspection of the data. Between‐group comparisons were made with chi‐squared tests, Student *t*‐tests or Mann–Whitney *U*‐tests, as appropriate.

## Results

### Overview

**Fig. 1:**
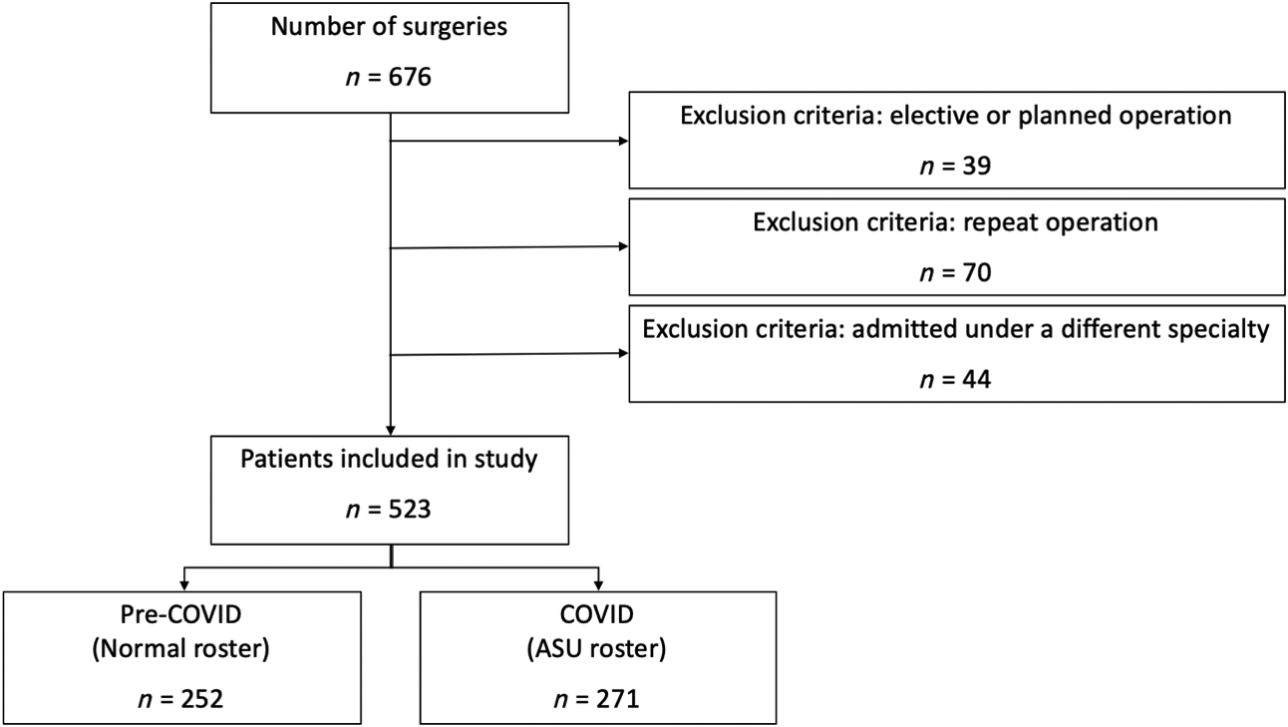
Flowchart of patients included in our retrospective study for data analysis.

During the specified four‐month time period, there were 969 surgical admissions under general and vascular surgery, and 676 emergency operations were performed. Overall, 54% of surgical admissions received operative management. After application of the exclusion criteria, 523 patients were admitted and operatively managed. There were 252 patients in the pre‐COVID‐19 group and 271 in the COVID‐19 group (Fig. 1); 65.7% of patients in the pre‐COVID‐19 group were booked for surgery on admission, compared with 68.3% in the COVID‐19 group.

### Population characteristics

The populations included in our study were comparable and heterogenous at baseline, with no statistically significant differences between age, sex or case acuity. There was a trend towards more unwell patients in the COVID‐19 group, as determined by American society of anaesthesiologists (ASA) classification (*P* = 0.046) (Table 1).

**Table 1:** Demographic data of included patients according to group

Characteristic	Pre‐COVID‐19 group (*n* = 252)	COVID‐19 group (*n* = 271)	Difference between groups (*P*‐value)*
Age			
Mean (*SD*)	38.9 (±21.6)	40.3 (±21.7)	NS† (*P* = 0.43)
Sex – no. (%)			NS (*P* = 0.95)
Female	129 (51.2)	137 (50.6)	
Male	123 (48.8)	134 (49.4)	
ASA classification – no. (%)			Yes, significant (*P* = 0.054)
1	74 (29.4)	56 (20.7)	
2	108 (42.9)	112 (41.3)	
3	57 (22.6)	79 (29.2)	
4	12 (4.8)	19 (7.0)	
5	1 (0.4)	5 (1.8)	
Case acuity – no. (%)			NS (*P* = 0.42)
Category A (<1 h)	2 (0.80)	4 (1.47)	
Category B (<4 h)	21 (8.33)	20 (7.38)	
Category C (<24 h)	208 (82.54)	233 (85.98)	
Category D (<10 days)	21 (8.33)	14 (5.17)	
Investigations in ED			
Biochemistry	222 (88.1)	230 (84.9)	NS (*P* = 0.34)
Radiology	121 (48.0)	134 (49.4)	NS (*P* = 0.81)

*Significance, *P* < 0.05. †NS, not significant at *P* < 0.05. ASA, American Society of anaesthesiologists physical status classification; *SD*, standard deviation.

### Surgical outcomes

There was a statistically significant difference in consultant surgeon presence between the two rosters, with a stronger consultant presence in theatre (*P* = 0.0001) and as primary operator (*P* = 0.02) in the COVID‐19 group (Table 2). There was no statistically significant difference between median case length, mortality within 30 days, return to theatre rates and complication rates (Table 2). The proportion of cases in‐hours compared with after‐hours or hospital LOS were identical between groups, despite increased operating capacity after hours (data not tabled). A comparison of most common operations for each roster can be seen in Table 3.

**Table 2:** Comparison of surgical outcomes between pre‐COVID on‐call rostering and COVID ASU rostering

Outcome	Pre‐COVID‐19 group (*n* = 252)	COVID‐19 group (*n* = 271)	Difference between groups (*P*‐value)*
Consultant surgeon – no. (%)			Yes, significant (*P* = 0.0001)
Present	88 (34.9)	140 (51.2)	
Available/not present	164 (65.1)	131 (48.8)	
Primary operator	22 (25)	56 (40)	Yes, significant (*P* = 0.02)
Median operation time	37 min (IQR: 17–77.25 min)	47 min (IQR: 21–85.5 min)	NS† (*P* = 0.054)
Mortality (within 30 days) – no. (%)			NS (*P* = 0.12)
Yes	1 (0.4)	5 (1.85)	
No	251 (99.6)	266 (98.15)	
Return to theatre – no. (%)			NS (*P* = 0.14)
Yes	17 (6.75)	28 (10.33)	
No	235 (93.25)	243 (89.67)	
Complication rate (within 30 days) – no. (%)			NS (*P* = 0.69)
Complication(s)	44 (17.46)	51 (18.82)	
No complication(s)	208 (82.54)	220 (81.18)	

*Significance, *P* <0.05. †NS, not significant at *P* <0.05. IQR, inter‐quartile range.

**Table 3:** Comparison of common operative cases between rosters

Diagnosis of cases requiring operative management	Pre‐COVID‐19 group(*n* = 252)	COVID‐19 group (*n* = 271)
General surgery		
Abscess	76	64
Acute cholecystitis	12	19
Appendicitis	77	75
Bowel obstruction	7	10
Bowel perforation	3	7
Carbuncle	11	7
Incarcerated hernia	8	8
Trauma laparotomy	3	3
Vascular surgery		
Debridement of diabetic foot infection/ulcer	16	14
Lower limb ischaemia	6	3
AAA rupture	0	2

### Surgical RAU

The RAU saw 92 patients over a 9‐week period, resulting in 31 operations from 34 admissions, which included 11 incision and drainage of abscesses and 12 laparoscopic appendicectomies (data not shown). Patients seen in the RAU accounted for ~11% of operations undertaken in this period and less than 10% of surgical admissions.

### NEAT targets

There was a statistically significant improvement in median NEAT times in the COVID‐19 ASU rostering between groups for ED workup time (69 versus 108 min), median surgical review time (61 versus 76.5 min) and median transport time (99 versus 106.5 min) (Table 4). However, only the ED workup time fell within NEAT standards. The proportion of surgical patients requiring emergency doctor review and investigations (pathology, radiology) were identical between the two groups (Table 1).

**Table 4:** Comparison of National Emergency Access Target (NEAT) times between pre‐COVID on‐call rostering and COVID ASU rostering

NEAT time	Pre‐COVID‐19 group (*n* = 252)	COVID‐19 group (*n* = 271)	Difference between groups (*P*‐value)*
Median Emergency Department workup time (<2 h until referral)	108 min (IQR: 47.75–182.25 min)	69 min (IQR: 33–129.5 min)	Yes, significant (*P* <0.00001)
Median surgical review time (1 h from ED referral)	76.5 min (IQR: 37.75–134 min)	61 min (IQR: 38.5–95 min)	Yes, significant (*P* = 0.00076)
Median transport time (1 h from surgical decision)	106.5 min(IQR: 72–170.25 min)	99 min (IQR: 58–158 min)	Yes, significant (*P* = 0.029)

*Significance, *P* <0.05. IQR, inter‐quartile range.

## Discussion

This study aimed to determine whether the emergency surgical workload for our general and vascular surgical departments changed during the initial height of the COVID‐19 pandemic, and whether our new ASU rostering during this time reduced NEAT times and correlated with improved patient outcomes.

### Population characteristics

Baseline characteristics of the populations were comparable, other than a trend towards more unwell patients during COVID‐19 (*P* = 0.046) (Table 1) as determined by ASA classification.^3^ The proportion of surgical patients undergoing investigations in ED (pathology, radiology) was the same between the two groups, despite earlier surgical contact (Table 1). Emergency surgical workload during this time did not change, and our numbers in the pre‐COVID and COVID groups were similar (252 and 271 patients respectively). This is consistent with data from a recently published Queensland study.^4^

### Surgical outcomes

Implementation of an ASU roster that emphasized theatre and on‐site consultant presence and eliminated rostering conflicts did significantly increase the level of consultant surgeon presence at surgical cases (*P* = 0.0001) and as the primary operator (*P* = 0.02) (Table 2), a phenomenon documented in other Australian studies.^5^ This would have been further impacted by reduced elective and private work.

Despite increased consultant operating, there was no significant difference in complication rates or overall surgical outcomes between the two patient groups. This supports the concept that patient outcomes and rates of complication are a product of pathology and the entire peri‐operative care package, rather than operative management alone.

There have been several published reports indicating an increase in delayed hospital presentations due to fear from contracting COVID‐19, resulting in more unwell patients at baseline and higher overall complication rates.^6^, ^7^, ^8^, ^9^ The ASA trend and complication rates seen in our study are also consistent with these findings.

There was no increased advocacy for non‐operative management at our hospital during the COVID period compared with pre‐COVID and this was evidenced by our reported case numbers.

### NEAT targets

The ASU rostering employed during COVID‐19 significantly improved all domains outlined in the NEAT scheme (Table 4). Less emergency department presentations and greater availability of surgical staff on ASU rostering allowed for faster surgical review and consultant‐led decision‐making, contributing to the reduction in NEAT times. Also, contributing were less internal referrals (data not shown) as well as reduced demands from elective surgery demands.

The greatest delay in a patient's journey through the emergency department was physical transport out of the department. This improved in the COVID‐19 group compared with pre‐COVID, largely due to a reduction in overall hospital workload, however, still far exceeds the NEAT target of 1 h. Therefore, despite mobilizing all facets of our surgical workforce, we were unable to meet our 4‐h NEAT target. This finding supports the need for a coordinated, hospital‐wide approach to improving emergency department in order to achieve national targets. However, it also brings into question the achievability and safety of the NEAT targets.

Evidence from the UK has demonstrated that achieving NEAT guidelines was expensive, did not improve time to assessment or mortality and did not result in a consistent improvement in care.^10^ An Australian study on NEAT targets conducted by urologists in Western Australia yielded similar results, with only a modest reduction in ED LOS and no improvement in time to theatre. They noted an increase in inappropriate referrals and an increase in inter‐unit transfer of undifferentiated patients who did not require specialist input.^11^ No hospital in Australia has consistently met NEAT targets >85%^12^ and clinicians need to be wary that standardized triage and referral targets do not compromise their patient care.

The implementation of a weekday ‘surgical rapid assessment unit’ (RAU) at our hospital was designed to bypass the emergency department for well patients with surgical pathology. However, this did not decrease the number of surgical patients being seen and assessed in the emergency department when compared with pre‐COVID‐19 numbers. The RAU service was appropriately resourced but underutilized as one of many new introductions during COVID‐19, an already stressful period of change for staff. This again emphasizes the importance of introducing structural changes in a coordinated manner with global support across hospital departments (Table 4).

This study is limited by the accuracy of recording of NEAT times in the electronic medical records, as contemporaneous medical records are not always maintained in an emergency environment. Data were collected manually to reduce administrative error, and it is likely that similar recording errors were made in both pre‐COVID and COVID data sets. There are also inherent limitations of a retrospective data set in a single hospital over a short time period, and this research could benefit from a follow‐up prospective study. It may also be interesting to compare manually collated data against administrative emergency department data for internal validity.

## Conclusion

The aim of this study was to determine whether emergency surgical workload at a busy regional hospital changed during the height of the COVID‐19 pandemic, and to assess the effect of an abundantly resourced surgical unit with a dedicated ASU and RAU on emergency department targets and patient outcomes. The data from this 4‐month retrospective analysis demonstrates that emergency surgical workload did not change, and that there was no change in overall patient outcomes or complication rates. Maximizing the availability of surgical staff for the emergency department with an ASU roster did result in a reduction of NEAT times, although, not to the designated national target time. The largest NEAT delay at our hospital consistently remains physical transport out of the emergency department, rather than delays in workup and surgical review, and this supports the need for a coordinated, hospital‐wide approach to improving flow. The overall feasibility and safety of NEAT times have also been highlighted again. We hope this research can contribute to improving the provision of surgical care in the ongoing COVID‐19 pandemic and beyond.

## Conflict of interest

None declared.

## Author contributions

**Jonathan H. Wiener:** Data curation; formal analysis; writing – review and editing. **Kate Swift:** Conceptualization; data curation; formal analysis; investigation; methodology; writing – review and editing. **Phuc (Richard) Do:** Data curation; writing – review and editing. **Roxanne Wu:** Conceptualization; methodology; project administration; supervision; writing – review and editing. **Matthew Corbitt:** Data curation; formal analysis; investigation; methodology; writing – review and editing; writing – review and editing.

## References

[1] Wiadji E , Mackenzie L , Reeder P *et al*. Utilization of telehealth by surgeons during the covid 19 pandemic in Australia: lessons learnt. ANZ J. Surg. 2021; 91: 507–14. 33634949 10.1111/ans.16693 PMC8013989

[2] Ang ZH , Wong S , Truskett P . General surgeons Australia's 12‐point plan for emergency general surgery. ANZ J. Surg. 2019; 89: 809–14. 31280492 10.1111/ans.15327

[3] American Society of Anesthesiologists (ASA) . ASA Physical Status Classification System. (2020).

[4] Fowler S , Zahir SF , Manning W , Kearney A , Sturgess D . Effect of the covid ‐19 pandemic first wave and public policy on elective and emergency surgery provision in southern Queensland. ANZ J. Surg. 2021; 91: 249–54. 33522697 10.1111/ans.16568 PMC8013170

[5] Beardsley CJ , Sandhu T , Gubicak S , Srikanth SV , Galketiya KP , Piscioneri F . A model‐based evaluation of the Canberra hospital acute care surgical unit: acute care surgery: a case of one size fits all? Surg. Today 2014; 44: 884–7. 24178132 10.1007/s00595-013-0775-2

[6] Solis E , Hameed A , Brown K , Pleass H , Johnston E . Delayed emergency surgical presentation: impact of corona virus disease ( covid‐19) on non‐covid patients. ANZ J. Surg. 2020; 90: 1482–3. 32437037 10.1111/ans.16048 PMC7280650

[7] Kamil AM , Davey MG , Marzouk F *et al*. The impact of COVID‐19 on emergency surgical presentations in a university teaching hospital. Ir. J. Med. Sci. [Epub ahead of print] 2021. 10.1007/s11845-021-02709-w. PMC8274665 34254230

[8] Ciarleglio FA , Rigoni M , Mereu L *et al*. The negative effects of COVID‐19 and national lockdown on emergency surgery morbidity due to delayed access. World J. Emerg. Surg. 2021; 16: 37. 34256781 10.1186/s13017-021-00382-z PMC8276199

[9] Winter Beatty J , Clarke JM , Sounderajah V *et al*. Impact of the COVID‐19 pandemic on emergency adult surgical patients and surgical services: an international multi‐center cohort study and department survey. Ann. Surg. 2021; 274: 904–12. 34402804 10.1097/SLA.0000000000005152

[10] Jones P , Schimanski K . The four hour target to reduce emergency department ‘waiting time’: a systematic review of clinical outcomes: the UK four hour rule. Emerg. Med. Australas. 2010; 22: 391–8. 20880296 10.1111/j.1742-6723.2010.01330.x

[11] Perera M , Gnaneswaran N , Roberts MJ , Lawrentschuk N , Ritchie P , Chan STF . Increased burden on metropolitan urological services: the era of the Australian National Emergency Access Targets (NEAT or the “4‐h target”). Urol Ann 2018; 10: 146–9. 29719324 10.4103/0974-7796.164843 PMC5907321

[12] Australian Institute of Health and Welfare . Australian hospital statistics 2013–14: emergency department care (Cat. No. HSE 153; Health Services Series No. 58). (2014).

